# Prognostic indicators in pituitary adenoma surgery: a comprehensive analysis of surgical outcomes and complications

**DOI:** 10.3389/fendo.2023.1327404

**Published:** 2024-01-11

**Authors:** George Riley, Nicolas Scheyer, Marc Klein, Isabelle Merlot, Bruno Guerci, Elodie Jeanbert, Lea Demarquet

**Affiliations:** ^1^ Endocrinology, Diabetes and Nutrition, Centre Hospitalier Universitaire de Nancy, Nancy, France; ^2^ Neurosurgery, Centre Hospitalier Universitaire de Nancy, Nancy, France; ^3^ Data Management and Statistics Unit, Centre Hospitalier Universitaire de Nancy, Nancy, France

**Keywords:** nonfunctioning pituitary adenoma (NFPA), pituitary adenoma (PA), endoscopic endonasal surgery (EES), adenoma, surgery complications and outcome, pronostic and predictive factors

## Abstract

**Objective:**

The primary aim of this study was to identify predictive factors associated with onset of *de-novo* clinically significant pituitary insufficiencies following endoscopic endonasal surgery (EES) for pituitary adenomas. The secondary objective explored the predictive factors of surgical success

**Methods:**

A retrospective analysis was conducted on 211 patients who underwent EES. Logistic regression models were employed for the primary and secondary objectives. Patients were stratified into specific groups based on surgical indications and prolactin levels for nuanced analysis.

**Results:**

Significant predictors for *de-novo* pituitary insufficiencies included male sex (OR 3.3, CI95% 1.3-8.1, p=0.01), immediate postoperative insufficiencies (OR 5.6, CI95% 2.8-11.1, p<0.001), and HYPRONOS criteria (OR 5.7, CI95% 1.6-20.9, p=0.008). For surgical success, preoperative insufficiencies (OR 0.7, CI95% 0.5-0.9, p=0.008), repeat surgeries (OR 0.1, CI95% 0-0.4, p=0.001), and gonadotroph or somatotroph adenomas were significant. Age and adenoma size were not predictive in multivariate analysis. Furthermore, we observed a “dip and recover” effect of prolactin after surgery and lower prolactin levels at follow-up (< 3 ng/ml) are correlated with more anterior pituitary insufficiencies than normoprolactinemic patients (p = 0.004).

**Conclusion:**

This study identifies key predictors for outcomes in pituitary surgery. Our research is the first to employ individualized success criteria for EES, challenging existing perceptions about the role of age and adenoma size. These findings open avenues for nuanced, individualized preoperative risk assessment and postoperative management.

## Introduction

Pituitary adenomas (PA), which constitute approximately 15% of all intracranial tumors (1), originate from the anterior lobe of the pituitary gland. These neoplasms are generally slow-growing and are in the vast majority of cases benign (2). They can be broadly classified into two categories: non-functioning pituitary adenomas (NFPAs) and secretory adenomas. Surgical intervention is often considered the first-line treatment for NFPAs that are associated with visual deficits or hypopituitarism (3, 4). Similarly, surgery is also recommended for most types of secretory adenomas, with the notable exception of prolactinomas (5). Modern pituitary surgery is performed by an endoscopic endonasal approach and is considered an effective treatment of PAs.

Complications arising from pituitary surgery are not uncommon and can be categorized into two primary types. The first category encompasses purely surgical complications, which include conditions such as meningitis (1-2% (6, 7)), meningeal breach (3-5% (6, 7)), and much more rarely intracranial hemorrhage. The second category pertains to endocrine-related complications affecting either the anterior or posterior lobe of the pituitary, leading to conditions like hypopituitarism (8-16% (8, 9)), most notably for surgical re-interventions (8, 9). Treatment of these conditions requires long term substitution and patient education through dedicated programs (10). Complications affecting the posterior pituitary, such as arginine-vasopressin (AVP) deficiency leading to central diabetes insipidus, are also recognized (6, 11). These complications are often transient in nature (6, 11).

Predicting the outcome of pituitary surgery, particularly the risk of complications, is a critical determinant in selecting the appropriate treatment modality for patients. To date, only a limited number of risk factors have been identified for complications following pituitary surgery, including tumor size, younger age, and previous surgical intervention (12, 13). Furthermore, existing studies have demonstrated that prolactin levels can serve as a surrogate marker for assessing pituitary function, specifically revealing associations between lower prolactin levels and anterior pituitary insufficiencies (14).

In this article, we present the findings of our retrospective study on the “HYP’OP” cohort, with the aim of identifying predictive factors for both surgical outcomes and complications.

## Methods

### Study population

The ‘HYP’OP’ cohort consists of patients who underwent endoscopic endonasal surgery (EES) for PA at the Nancy University Hospital during a 10-year period from January 2012 to June 2022. As part of the standard care protocol, post-operative hospitalization in our endocrinology department is routine, followed by a systematic reevaluation 4-6 months after the surgical intervention. Approximately half of the patients in this cohort were hospitalized in our department for post-operative evaluation, while the remaining were referred to other hospitals for their post-operative care. Inclusion criteria for this study were as follows: 1) having undergone EES for PA at Nancy University Hospital within the specified timeframe, 2) post-operative hospitalization and follow-up care in our endocrinology department, and 3) surgery performed by one of the two neurosurgeons who conduct over 50 interventions per year. Only patients who met all these criteria were included in the study.

### Definitions

NFPA was defined as the presence of a sellar mass, sellar masses that lack clinical and biochemical evidence of hormone hypersecretion.

Secreting adenomas were classified based on their specific type of secretion. Concordance between anatomopathological reports and biological findings was required in all cases. Lactotroph adenomas were defined as secreting if prolactin levels exceeded 100 ng/ml and were supported by concordant anatomopathological findings. Somatotroph adenomas were classified as secreting if patients exhibited overt signs of acromegaly or elevated GH/IGF-1 levels both before and persisting after an oral glucose tolerance test. Mammosomatotroph adenomas had to meet both the lactotroph and somatotroph criteria to be considered secreting. Corticotroph adenomas were considered secreting if patients displayed overt signs of Cushing’s disease or persistent hypercorticism following a standard oral dexamethasone suppression test. Secreting gonadotroph and thyrotroph adenoma were not observed in our cohort.

Insufficiencies were defined based on the specific hormonal axis involved. Growth hormone deficiency was identified by an IGF-1 level below our laboratory’s reference range, excluding patients with altered liver function. Adrenal insufficiency was defined by a basal cortisol level at 8 am lower than 5 µg/dl (138 nmol/l) or lower than 18 µg/dl (500 nmol/l), coupled with an absence of response to a 250µg ACTH stimulation test (SST). Central hypothyroidism was characterized by low free T4 levels and low or normal TSH levels, in accordance with our laboratory reference ranges. Central hypogonadism was defined by low total testosterone or low estradiol levels in conjunction with normal or low LH levels. In women under the age of 51, the average menopausal age in France, this condition was considered “clinically significant”. AVP deficiency was defined by the requirement for oral desmopressin treatment, which was determined based on observations of altered urine concentration. Diagnostic criteria were established either through static tests or dynamic water deprivation tests, depending on the individual patient’s condition.

### Data collection

Data were retrospectively collected on all included patients at four time points: preoperatively, postoperatively (within 5 days following surgery), during the follow-up hospitalization (4 – 6 months after surgery), and at the last known follow-up date. Anatomopathological data were also collected.

Preoperative data collection encompassed evaluations of each hormonal axis, the corresponding treatment status for each specific axis, and visual assessments—most commonly through Goldmann field tests. Additionally, pituitary magnetic resonance imaging (MRI) data were gathered, detailing factors such as adenoma size, optic nerve compression and invasion of the cavernous sinus.

Postoperative data collection included evaluations of each hormonal axis and assessments of surgical-related complications, such as meningitis, meningeal breach, intracranial hemorrhage, post-operative diabetes insipidus and post-operative syndrome of inappropriate antidiuretic hormone secretion (SIADH).

Follow-up data comprised evaluations of each hormonal axis and treatment status, along with a follow-up MRI specifically assessing residual tissue and persistent optic nerve compression.

Anatomopathological data collected included the Ki67 proliferation index, the status according to HYPOPRONOS proliferation criteria (using Ki67, mitoses and p53 status) as defined by Trouillas et al. (15), and the results of immunohistochemical staining.

### Objectives

The **primary objective** of this study was to assess the risk factors associated with complications arising from EES, specifically targeting the incidence of *de-novo*, clinically significant pituitary insufficiencies. The endpoint was assessed at the 4-month follow-up and was defined as a composite criterion comprising any of the following: central adrenal insufficiency (excluding patients with Cushing’s disease), clinically significant central hypogonadism (in female patients age had to be lower than 51), growth hormone deficiency (excluding patients with acromegaly), central hypothyroidism, or AVP deficiency. Importantly, all these complications had to be ‘*de-novo*’ and not present prior to the surgical intervention.

**The secondary objective** of this study was to evaluate factors predictive of the ‘success’ of EES. Given the nuanced nature of ‘success,’ patients were stratified into five groups based on surgical indications: Group 1 comprised non-secreting, non-compressive adenomas without preoperative insufficiency or hyperprolactinemia, with success defined as no new clinically significant insufficiency and no MRI-observed residual tissue. Group 2 included non-secreting, non-compressive adenomas without preoperative insufficiency but with disconnection hyperprolactinemia, with success defined as no new clinically significant insufficiency and regression of hyperprolactinemia. Group 3 involved non-secreting adenomas with at least one clinically significant preoperative axis deficiency, regardless of disconnection hyperprolactinemia, with success defined as restoration of at least one deficient axis without new clinically significant insufficiency. Group 4 consisted of secreting adenomas, with success defined as regression of hypersecretion. Group 5 included adenomas operated on solely for their compressive nature on the optic pathways, with or without disconnection hyperprolactinemia, with success defined as no compression observed on the follow-up MRI.

In all groups, if the patient underwent reoperation or received radiotherapy postoperatively, the surgery was not considered successful. Patients not falling in to one of the five groups were manually attributed to a group depending on surgical indication.

### Statistical analysis

Characteristics of samples were described by percentage for categorical variables and mean, standard deviation, median, quartiles and min/max values for continuous variables.

For both the **primary objective and secondary objective**, logistic regression models were employed to assess the risk factors associated with the incidence of *de-novo*, clinically significant pituitary insufficiencies at the 4-month follow-up and to evaluate the factors predictive of the ‘success’ of EES, as defined by the specific endpoints for each group. Variables with a p-value less than 0.2 in bivariate analysis were eligible for the multivariable model. A stepwise selection procedure was applied to retain significant independent factors. Associations were described by odds ratios (OR) and 95% confidence intervals (CI).

The significance threshold was set at 5%. These statistical analyses were performed using SAS version 9.4 software (SAS Institute).

## Results

### Patient demographics, clinical presentation and tumor characteristics

Over the 10-year period, there were 211 cases that met the inclusion criteria after excluding 2 patients that were opposed to participation in the study and after excluding non-adenoma pituitary tumors. The baseline characteristics of these patients are presented in **Table 1**.

**Table 1:** Demographics, clinical presentation, and tumor characteristics of 211 patients before surgery, or less if specified by n.

Value	N(%)/ Median (min-max)
Age (years)	54.0 (18.0 – 86.0)
Female sex	114 (54.0%)
First surgery	186 (88.2%)
**Tumor size (mm)**	21.0 (2.6 – 83.0)
Microadenoma (<10 mm)	34 (20.5%)
Macroadenoma (10-40 mm)	170 (80.6%)
Giant adenoma (>40 mm)	7 (3.3%)
Ophthalmic symptoms	76 (36.4%)
Optical compression on MRI	114 (54.3%)
Cavernous invasion on MRI	80 (39.0%)
Anatomopathology	
Gonadotroph adenoma	91 (43.1%)
Corticotroph adenoma	27 (12.8%)
Lactotroph adenoma	29 (13.7%)
Somatotroph adenoma	17 (8.1%)
Thyrotroph adenoma	0 (0.0%)
Mixed adenoma	10 (4.7%)
Mammosomatotroph adenoma	8 (3.8%)
Non-contributive	18 (8.5%)
Missing or impossible during surgery	19 (9.0%)
Ki67-index %	2.1 (0.0 – 12.0)
Proliferant HYPOPRONOS status	19 (10.2%)
Gonadal axis status (n=200)	
Insufficiency	70 (35.0%)
Hypergonadism	0 (0.0%)
Thyroid axis status (n=202)	
Insufficiency	46 (22.7%)
Central hyperthyroidism	0 (0.0%)
HPA axis status (n=201)	
Insufficiency	33 (16.4%)
Cushing's disease	24 (12.0%)
With treatment	13 (6.5%)
Prolactin axis status (n=199)	
Hyperprolactinemia	76 (38.2%)
Disconnection hyperprolactinemia	47 (66.2%)
Lactotroph adenoma with hyperprolactinemia	24 (33.8%)
With treatment	14 (6.7%)
GH axis status (n=198)	
Insufficiency	22 (11.1%)
Acromegaly	30 (15.2%)
With treatment	12 (6.1%)
At least one insufficiency	89 (43.6%)

MRI, Magnetic Resonance Imaging; HPA, Hypothalamic-Pituitary-Adrenal; GH, Growth Hormone.

The median age was 54,0 years, and 114 (54,0%) patients were female. The median tumor size was 21,0 mm (ranging from 2.6 – 83,0 mm) with 170 (80.6%) of tumors between 10-40 mm. Ophthalmic symptoms and optical compression on MRI were present in 76 (36.4%) and 114 (54.3%) patients respectively. Cavernous invasion was observed in 80 (39,0%) of patients.

The majority of tumors were gonadotroph adenomas (43.1%), all were non-functioning. Corticotroph adenomas represented 12.8% of cases (27 patients, of which 24 presented overt Cushing’s syndrome, 13 requiring treatment). Lactotroph adenomas represented 13.7% of cases (29 patients, 24 of which presented hyperprolactinemia, 14 requiring treatment). Somatotroph adenomas represented 8.1% of cases with an additional 3.8% being mammosomatotroph adenomas (25 patients in total, 12 of which required treatment). No thyrotroph adenoma were identified.

At least one pre-surgical insufficiency was observed in 89 patients (43.6%), most frequently gonadal insufficiency (35,0%), followed by central thyroid insufficiency (22.7%), secondary adrenal insufficiency (SAI) (16.4%) and GH insufficiency (11.1%). Five patients presented with an AVP deficiency before surgery (3 of which had never had EES).

### Post-operative and follow-up evaluation characteristics

The follow-up evaluation was conducted at a median of 136,5 days (Q1-Q3: 123,0-158,0) postoperatively. Data is summarized in **Table 2**.

**Table 2:** Post-operative and follow-up data for 211 patients.

Value	Post-operative evaluation	Follow-up evaluation
Days after surgery as median (Q1-Q3)	–	136.5 (123.0 – 158.0)
Gonadal axis status (n=204 & n=203)		
Insufficiency	97 (47.5%)	75 (36.9%)
New insufficiency (vs pre-operative)	33 (26.0%)	25 (19.5%)
Recovery (vs pre-operative)	–	18 (25.7%**)
Not evaluated or missing	7	8
Thyroid axis status (n=206 & n=200)		
Insufficiency	67 (32.5%)	64 (31.0%)
New insufficiency (vs pre-operative)	25 (16.6%)	26 (17.5%)
Recovery (vs pre-operative)	–	10 (21.7%**)
Not evaluated or missing	5	11
HPA axis status (n=111 & n=204)		
Hypersecretion	5 (4.5%)	8 (5.0%)
Newly identified (vs pre-operative)	0 (0.0%)	1 (0.6%)
Insufficiency	29 (26.1%)	45 (22.1%)
New insufficiency (vs pre-operative) †	14 (17.1%)	21 (15.1%)
Recovery (vs pre-operative)	–	14 (42.4%**)
Not evaluated or missing	100	7
That were normal at follow-up	–	69 (69.0%)
GH axis status (n=210 & n=205)		
Hypersecretion	27 (12.9%)	23 (11.2%)
Newly identified	5 (2.9%)	1 (0.6%)
Insufficiency	31 (14.8%)	29 (14.1%)
New insufficiency (vs pre-operative) †	15 (10.3%)	15 (10.5%)
Recovery (vs pre-operative)	–	10 (45.5%**)
Not evaluated or missing	1	6
At least one new insufficiency	–	62 (32.6%)
Regression of a pre-op hypersecretion	–	34 (45.9%)
Recovery of at least one axis	–	45 (50.6%)
Arginine-vasopressin deficiency	29 (13.7%)	19 (9.2%)
Recovery (vs pre-operative)	–	4 (80.0%**)
SIADH	1 (0.5%)	0
Meningitis	5 (2.4%)	–
Meningeal breach	7 (3.3%)	–
Intracranial hemorrhage	2 (0.9%)	–

† represents data where patients with a hypersecretion of that axis were excluded. **, percentage of initially insufficient patients; SIADH, syndrome of inappropriate antidiuretic hormone release; HPA, Hypothalamic-Pituitary-Adrenal; GH, Growth Hormone. Prolactin data represented separately.

Data presented as number (percentage of available data) or as median (Q1-Q3) when applicable.

Gonadal axis insufficiency was noted in 97 patients (47.5%) postoperatively, declining to 75 (36.9%) at follow-up. New cases were observed in 33 patients (26,0%) initially and 25 (19.5%) at follow-up. 18 patients (25.7%) previously presenting hypogonadism had recovered by the follow-up evaluation.

Thyroid insufficiency was observed in 67 patients (32.5%) postoperatively, with a slight decrease to 64 patients (31,0%) at follow-up. New cases of thyroid insufficiency were observed in 25 patients (16.6%) postoperatively and 26 (17.5%) at follow-up (17.5%). 10 patients (21.7%) with initial central hypothyroidism recovered by the follow-up evaluation.

Cortisol hypersecretion was noted in 5 patients (4.5%) postoperatively and 8 patients (5,0%) at follow-up, with one new case due to the activation of a previously silent corticotroph adenoma. Postoperative cortisol insufficiency was seen in 29 patients (26.1%), proportionally decreasing to 45 patients (22.1%) at follow-up. New insufficiencies were identified in 14 patients (17.1%) postoperatively and 21 patients (15.1%) at follow-up. Data was missing or unevaluated for 100 patients (47.4%), of whom 69 (69%) normalized at follow-up. 14 patients (42.4%) presenting SAI before surgery, recovered by the follow-up evaluation.

GH axis hypersecretion was found in 27 patients (12.9%) postoperatively, decreasing to 23 (11.2%) at follow-up. New instances were 5 (2.9%) postoperatively and 1 at follow-up. Insufficiency was observed in 31 patients (14.8%) postoperatively and 29 (14.1%) at follow-up. New insufficiencies were consistent at 15 patients (10.3% initially, 10.5% at follow-up). Notably, 10 patients exhibited GH deficiency at both evaluation points and 10 patients (45.5%) recovered from a pre-operative insufficiency at the follow-up point.

In total, 62 patients (32.6%) developed new axis insufficiencies postoperatively, which were not present pre-operatively. Additionally, 34 patients (45.9%) experienced a reduction in pre-operative hypersecretion (normal or low at follow-up), while 45 patients (22.1%) showed recovery of at least one axis insufficiency.

Surgical complications were rare, AVP deficiency was observed in 29 patients (13.7%) postoperatively, which reduced to 19 patients (9.2%) at follow-up. Interestingly, 4 of the 5 patients diagnosed with pre-operative AVP deficiency recovered. SIADH was noted in just one patient and was transient in nature. Meningitis, meningeal breach and intracranial hemorrhage were all rare events.

### Prolactin groups over time

The data showed a postoperative shift from normal prolactin levels to moderately low and severely low levels. This temporal decline in prolactin levels was followed by a partial recovery at the follow-up evaluation. This “dip-and-recovery” can be well observed in the prolactin levels shown in **Table 3**, this pattern suggests that pituitary surgery initially results in a decrease in prolactin levels, moving patients from a normoprolactinemic state into lower prolactin categories, with some reversion to normal levels over time.

**Table 3:** Prolactin group distribution depending on evaluation point, data presented as number (percentage).

Value	Pre-operative (n=176)	Post-operative (n=208)	Follow-up (n=194)
Group 1 (< 3 ng/ml)	2 (1.1%)	18 (8.7%)	8 (4.1%)
Group 2 (3 – 5 ng/ml)	7 (4.0%)	17 (8.2%)	8 (4.1%)
Group 3 (5 – 20 ng/ml)	73 (41.5%)	112 (53.8%)	113 (58.2%)
Group 4 (> 20 ng/ml)	51 (29.0%)	20 (9.6%)	22 (11.3%)
No sub-group (Prolactinomas or treated)	43 (24.4%)	41 (19.7%)	43 (22.2%)

43 patients excluded presenting lactotrope adenoma or treatment by prolactin altering medication.

Furthermore, an *ad-hoc* analysis using pairwise comparisons revealed a significantly higher incidence of anterior pituitary insufficiencies in patients with the lowest prolactin levels (< 3 ng/ml) compared to normoprolactinemic patients (5-20 ng/ml) (p=0.004).

### Primary objective

For the primary objective, which focuses on identifying risk factors associated with *de-novo* clinically significant pituitary insufficiencies at the 4-month follow-up, bivariate regression analysis identified several candidate variables. These included age (p=0.126), male sex (p=0.032), number of preoperative insufficiencies (p=0.113), macroadenoma type (p=0.049), initial cavernous sinus invasion detected on MRI (p=0.158), number of new immediate postoperative insufficiencies (p<0.001), immediate postoperative AVP deficiency or SIADH (p<0.001), postoperative surgical complications (p=0.01), Ki67 index (p=0.068), and proliferation according to HYPRONOS criteria (p=0.078).

**Figure 1:**
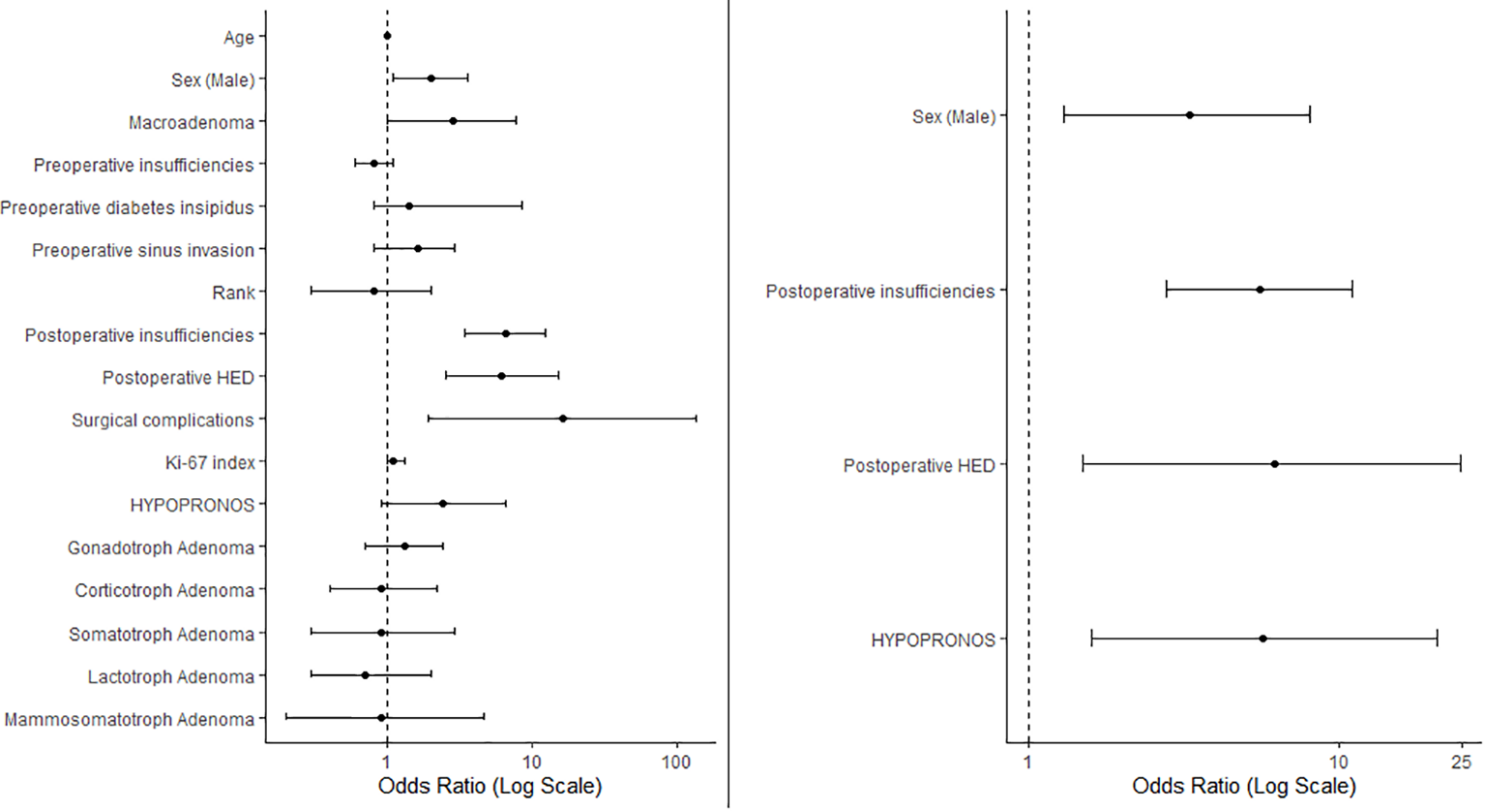
The left panel presents a forest plot of the univariate regression, illustrating the odds ratios (ORs) for various variables in relation to *de novo* clinically significant pituitary insufficiencies of the 4-month follow up. The right panel displays variables-retained after stepwise selection in the multivariate regression analysis. Data are represented as ORs with 95% confidence intervals. Rank, not first time surgery; HED, hydroelectrolytic disorders.

Following stepwise variable selection and multivariate regression analysis, the variables that remained significant were male sex (OR 3.3, CI95% 1.3-8.1, p=0.01), the number of new immediate postoperative insufficiencies (OR 5.6, CI95% 2.8-11.1, p<0.001), immediate postoperative AVP deficiency or SIADH (OR 6.2, CI95% 1.5-24.9, p=0.01), and proliferation according to HYPRONOS criteria (OR 5.7, CI95% 1.6-20.9, p=0.008). Results are summarized in **Figure 1**.

### Secondary objective

For this objective, which focuses on identifying factors associated with success of EES, bivariate regression analysis identified the following candidate variables: age (p=0.01), number of preoperative insufficiencies (p=0.082), lower pre-operative prolactin levels (p=0.1616), not first time surgery (i.e. rank of surgery > 1)(p=0.001), number of new immediate postoperative insufficiencies (p=0.178), immediate postoperative AVP deficiency or SIADH (p=0.027), gonadotroph adenoma subtype (p=0.006), somatotroph adenoma subtype (p=0.012) and mammosomatoroph adenoma subtype (p=0.078).

Following stepwise variable selection and multivariate regression analysis, the variables that remained significant were number of preoperative insufficiencies (OR 0.7, CI95% 0.5-0.9, p=0.008), not first time surgery (OR 0.1, CI95% 0-0.4, p=0.001), gonadotroph adenoma subtype (OR 3.4, CI95% 1.6-7.5, p=0.002) and somatotroph adenoma subtype (OR 5.3, CI95% 1.4-20.8, p=0.017).

**Figure 2:**
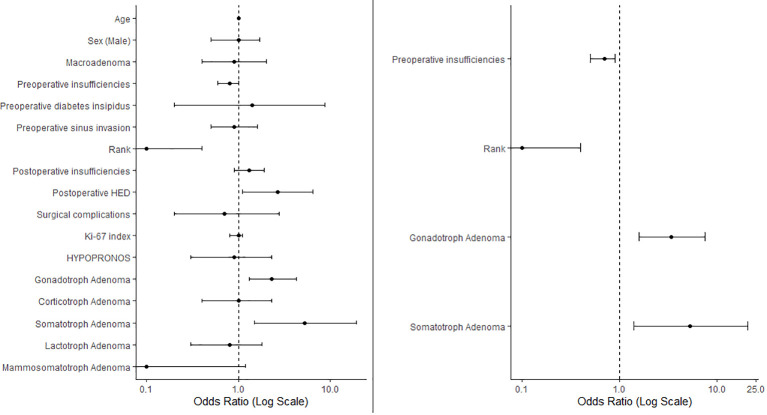
The left panel presents a forest plot of the univariate regression, illustrating the odds ratios (ORs) for various variables in relation to EES success. The right panel displays variables retained after stepwise selection in the multivariate regression analysis. Data are represented as ORs with 95% confidence intervals. ORs < 1 indicate negative predictors of surgical success. Rank, not first time surgery; HED, hydroelectrolytic disorders.

Results are summarized in **Figure 2**.

## Discussion

### Patient population and surgical outcomes

Baseline characteristics of patients are similar to what others describe, such as the nationwide population base study by Agustsson et al., published in 2015 (16). A noticeable difference is prolactinoma frequency which is evaluated at 40% in their study, vs 13.7% in ours (17.5% including mammosomatotroph adenomas). Other studies however also find lower values (7–9). The baseline hormonal characteristics of patients were also in line with other studies (17, 18). No cases of hypergonadotrophic hypergonadism or central hyperthyroidism were observed, which is expected considering their extreme rarity (19, 20).

Hormonal complications of the surgery were in line with other studies except hypocortisolism at follow-up at 15.1%, slightly lower than some other studies (21).

The recovery rates of preoperative insufficiencies when observed at the 4-month follow-up were variable depending on the affected axis ranging from a 45% recovery rate in GH insufficiency to a 22% recovery rate in central hypothyroidism. This data is similar to that reported in other studies (22). Webb et al. (21) describe 48% of patients recovering at least one axis, our results are very similar with recovery in 45 patients (50.6%).

Four out of the 5 patients (80%) exhibiting pre-surgical AVP deficiency recovered, this high rate of recovery raises questions about the accuracy of the initial diagnosis. Purely surgical complications were in line with previous studies except AVP deficiency which was slightly higher (7, 11).

### Primary objective and secondary objective

These objectives aimed to define factors associated complications, more specifically *de-novo* clinically significant pituitary insufficiencies, and success of pituitary surgery.

In some studies, age emerges as a risk factor for postoperative *de-novo* pituitary insufficiencies, a pattern also evident in our univariate analysis. However, our study is unique in analyzing an extensive range of variables utilizing a stepwise selection method post-bivariate analysis, in this context, age ceases to be an independent risk factor. This may indicate that the prognostic outcomes are more influenced by factors inherently associated with age rather than age per se.

In our study, male sex is observed as a risk factor for *de-novo* clinically significant hypopituitarism. This finding remains consistent even when the criteria for clinically significant central hypogonadism in women, specifically the age criterion, are excluded from consideration. The enduring significance of male sex in the multivariate models suggests that this risk is not contingent on tumor characteristics or type. While a smaller study by Harary et al. (18) also noted a similar trend, the significance of male sex did not withstand multivariate analysis in their findings.

We observed a strong correlation between immediate postoperative insufficiencies and *de-novo* clinically significant pituitary insufficiencies at the 4-month follow-up, while this underscores the importance of rigorous perioperative monitoring, this relationship also raises questions about the true prognostic value of these immediate insufficiencies. Given that the majority of follow-up insufficiencies are already deficient in the immediate postoperative period, these early insufficiencies may serve less as predictors and more as early manifestations of what frequently evolves into long-term pituitary dysfunction. *Ad hoc* analysis showed only 29 patients (34%) recover from a *de-novo* post-op insufficiency. Therefore, describing these immediate postoperative insufficiencies as “predictive” may be somewhat misleading; they might be more accurately termed “early indicators” of likely persistent pituitary dysfunction. In subsequent research, we aim to explore the reversibility of immediate postoperative insufficiencies and ascertain factors promoting recovery.

Unexpectedly, the presence of macroadenomas (>10mm) was significant only in bivariate analysis and did not retain its significance in multivariate analysis. Conversely, smaller microadenomas did not show a correlation with increased surgical success. This suggests that the size of the adenoma, while initially appearing to be a risk factor or predictor in bivariate analysis, may not independently influence surgical outcomes when other variables are considered. The absence of significance in multivariate analysis indicates that other confounding variables may overshadow or mitigate the impact of adenoma size on surgical success and complications. Interestingly, in a large meta-analysis by Joshi et al (23) which primarily investigated AVP deficiency after EES, multivariate analysis found tumor size as a risk factor for functional PA but not for NFPA. These findings suggest that the influence of tumor size on surgical outcomes may necessitate a more individualized approach, potentially accounting for the functional status of the adenoma.

Our findings indicate that postoperative hydroelectrolytic disorders serve as markers for persistent significant pituitary insufficiencies in multivariate analyses, as well as indicators of surgical success in bivariate analyses only. This could imply that such disorders are indicative of aggressive surgical approaches, which may yield both increased complications and higher rates of surgical success, and should be explored in further studies.

The substantial significance of HYPRONOS criteria in our study not only supports its established role in assessing long-term relapse risk (15) but also suggests its utility in predicting long-term complications. Particularly in instances where HYPRONOS criteria reveal aggressive adenoma behavior, their application could extend to inform more targeted therapeutic strategies and vigilant monitoring for potential complications. Thus, our findings suggest the need for further studies to explore an expanded role for HYPRONOS criteria in the comprehensive postoperative and long-term management of patients, specifically in predicting complications as well as relapse risk.

Finally, the identification of gonadotroph and somatotroph adenoma subtypes as significant predictors of EES success in the postoperative period offers valuable insights for long-term patient management.

### Prolactin “dip and recover”

Our study reveals a postoperative transition from normative prolactin concentrations to moderately and severely reduced levels, which exhibit partial recovery during subsequent follow-up. To the best of our knowledge, this is the first documentation of such a phenomenon outside of studies focused on prolactinomas that were excluded from our analysis. We also found that lower prolactin levels at follow-up are correlated with anterior pituitary insufficiencies, supporting the idea that prolactin levels could be employed as a marker for hypopituitarism in long-term follow-up. In future research we aim to model temporal variations in prolactin levels to offer a method for predicting pituitary insufficiencies.

### Study strength

The principal strength of this study lies in its comprehensive dataset, which includes a large cohort of 211 patients with complete follow-up, anatomopathological, and radiological data. This comprehensive dataset facilitated the individualized tailoring of success criteria for EES based on pre-operative characteristics. This novel approach not only challenges traditional metrics but also sets the stage for future research by providing a nuanced framework for both preoperative risk assessment and postoperative management.

### Study limitations

The main limitation of this study is its retrospective design, which may introduce bias and limit causal inference. Another limit was the lack of standardized criteria for AVP deficiency, this is particularly observed in the 80% recovery rate after surgery, suggesting potential errors in pre-surgical assessment. Furthermore, the sample size may have resulted in limited statistical power, thereby affecting the significance of variables when transitioning from bivariate to multivariate analysis. Consequently, future studies with larger sample sizes may be necessary to confirm or refute our findings. Additionally, the study was conducted at a single center, which may limit the generalizability of the findings.

It is also important to note that the evaluation of residual tumor as a criterion for surgical success was intrinsically linked to the specific surgical indications (groups), with considerations for residual tumor being particularly pertinent in groups where complete removal or decompression was the primary goal (groups 1 and 5). This aspect of the study design could affect the interpretation of surgical success across different groups.

## Conclusion

In conclusion, our study sheds light on key factors that influence the outcomes and complications of pituitary surgery, notably the onset of *de-novo* clinically significant pituitary insufficiencies. We find that male sex, immediate postoperative insufficiencies, and specific criteria such as HYPRONOS hold significance for predicting *de-novo* pituitary insufficiencies. Regarding predictors of successful surgical outcomes, our findings suggest that gonadotroph or somatotroph adenoma subtypes are positively correlated with successful interventions. In contrast, preoperative pituitary insufficiencies and instances where the surgery is not the patient’s first are indicative of an increased risk of surgical failure.

To the best of our knowledge, our research is the first study to employ individualized success criteria for EES based on pre-operative characteristics on a large scale. Each patient was evaluated in accordance with these personalized objectives. Under these conditions, our findings challenge some existing perceptions, like the role of age and adenoma size on surgical outcomes, opening avenues for more nuanced, individualized preoperative risk assessment and postoperative management strategies. The results also signal the need for further research to validate these factors and explore their broader applications in the comprehensive care of patients undergoing pituitary surgery.

## Data availability statement

The raw data supporting the conclusions of this article will be made available by the authors, without undue reservation.

## Ethics statement

This study adheres to the French MR004 regulatory framework for the processing of personal health data for research purposes. The study received ethical approval from the Institutional Review Board (IRB) of the Nancy University Hospital, with the approval number 2022PI198. In compliance with the French MR004 framework, all patients included in this study were informed of their participation and were provided with the opportunity to oppose their inclusion. As per MR004 guidelines, formal written informed consent was not required. All data were anonymized to protect patient confidentiality. The clinical trial is registered under the identifier NCT06053437.

## Author contributions

GR: Conceptualization, Data curation, Investigation, Resources, Visualization, Writing – original draft. NS: Validation, Writing – review & editing. MK: Writing – review & editing. IM: Writing – review & editing. BG: Supervision, Writing – review & editing. EJ: Methodology, Validation, Writing – review & editing. LD: Conceptualization, Supervision, Validation, Writing – review & editing.
